# Fungal Guttation, a Source of Bioactive Compounds, and Its Ecological Role—A Review

**DOI:** 10.3390/biom11091270

**Published:** 2021-08-25

**Authors:** Adam Krain, Piotr Siupka

**Affiliations:** Faculty of Natural Sciences, Institute of Biology, Biotechnology and Environmental Protection, University of Silesia in Katowice, 40-032 Katowice, Poland; adam.krain@us.edu.pl

**Keywords:** fungal guttation, exudates, ecological relationships, secondary metabolites, antimicrobials, peptaibols, destruxins, biocontrol agent, anticancer agents, proteins

## Abstract

Guttation is a common phenomenon in the fungal kingdom. Its occurrence and intensity depend largely on culture conditions, such as growth medium composition or incubation temperature. As filamentous fungi are a rich source of compounds, possessing various biological activities, guttation exudates could also contain bioactive substances. Among such molecules, researchers have already found numerous mycotoxins, antimicrobials, insecticides, bioherbicides, antiviral, and anticancer agents in exudate droplets. They belong to either secondary metabolites (SMs) or proteins and are secreted with different intensities. The background of guttation, in terms of its biological role, in vivo, and promoting factors, has been explored only partially. In this review, we describe the metabolites present in fungal exudates, their diversity, and bioactivities. Pointing to the significance of fungal ecology and natural products discovery, selected aspects of guttation in the fungi are discussed.

## 1. Introduction

Filamentous fungi produce a wealth of bioactive metabolites, many of which are considered important from the human point of view. Among them, toxic compounds are naturally produced, such as mycotoxins, cytotoxic agents, and carcinogens, as well as potentially useful molecules, including antibiotics, fungicides, insecticides, and antineoplastic or antiviral agents [1,2]. These compounds belong to secondary metabolites (SMs)—metabolites that are not engaged in the internal economy of the producing organism, but are essential for adaptation to specific environments, especially in situations that reflect survival probability [3,4]. Fungal SMs play an important role in protection from UV light damage, communication and signaling, defense against toxic molecules, and creating biological interactions—competition, symbiosis, or pathogenicity [2,4]. Importantly, SMs can be retained inside the cells or secreted into the surrounding environment, as well as exuded by guttation [5,6].

Guttation is a well-known phenomenon that involves the active exudation of water and dissolved substances, without tissue injury [7,8]. The formation of guttation droplets, which is primarily known from plants, is also widely observed among fungi [9,10,11]. Its ecological role, however, was undervalued and ignored in research for a long time. The first study on its putative functions was provided by McPhee and Colotelo [12]. They suggested that guttation enables the accumulation of metabolite reserves and removal of SMs or toxic byproducts of metabolism [12]. It was also suggested that the guttation process is linked to mycelium maturation [13], and that formation of exudates might serve as a water reservoir, allowing constant growth of aerial hyphae from afar (of their substrates) [14]. Deeper research into the composition and biological function of exudates have revealed that they may contain a wide range of bioactive compounds [5,15].

Here, we review reports on biologically active substances found in fungal exudates, with emphasis on molecules that have potential for practical applications, which are significant for human health and safety, and for uncovering putative roles of the guttation phenomenon in filamentous fungi. We also briefly summarize past and present views on fungal guttation from an ecological perspective, and conditions that promote secretion of guttation droplets. We provide an overview on this fascinating topic, which has still not been fully explored.

## 2. Conditions and Factors Promoting Guttation

**Figure 1:**
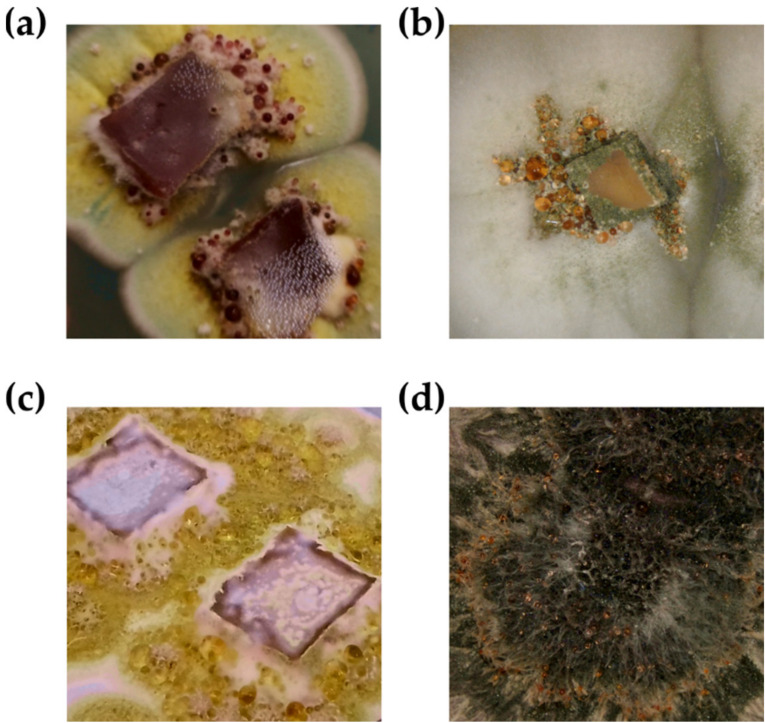
Fungal guttation—liquid droplets exuded on the fungi colony surface. (**a**–**c**) are the different strains belonging to *Aspergillus* genus; (**d**) strain belonging to *Gliomastix* genus.

**Figure 2:**
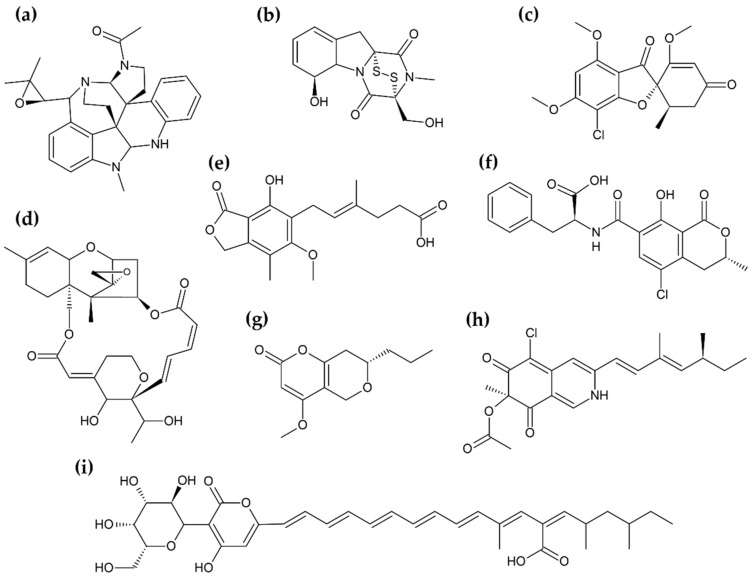
The structural diversity of SMs reported in fungal exudates. (**a**) communesin A; (**b**) gliotoxin; (**c**) griseofulvin; (**d**) macrocyclic trichothecene (satratoxin H); (**e**) mycophenolic acid; (**f**) ochratoxin A; (**g**) phomopsinone A; (**h**) sclerotioramine; (**i**) epipyrone A.

Droplet exudation occurs during defined external conditions; it is different for every fungus and appears only during a certain period of culture duration. Although guttation is observed in nature, laboratory culture is most convenient for the elucidation of all promoting factors and parameters [10,15,16]. Fungal guttation is an “everyday experience” when practicing mycology (Figure 1); standard culture media are often suitable to induce the phenomenon [14,16]. Among the studies focused on exudate investigations, the following media were utilized: potato dextrose agar [17,18], malt extract agar [19,20], and potato sucrose agar [21]. These were rarely specialized media, and involved synthetic, natural, and combined [15,22,23,24]. Hutwimmer et al. [15] indicated how big impact on a fungal guttation has the composition of sugars in growth medium. The study showed that a combination of more than one carbon sources, well-metabolized, and non-preferred sugars, could promote exudation [15]; thus, nutritional condition establishments were important in the experimental design. Culture temperature was of similar importance to culture media composition. The temperature range in which guttation occurred, most often, was from 20 °C to 30 °C [10,25,26]. A short summary of the described cultivation parameters, as well bioactive compounds found in fungal exudates, is presented in Table 1. The structural diversity of secondary metabolites found in the exudates is presented in Figure 2.

In some cases, specific factors are required to trigger guttation. They act by introducing a necessary signal, e.g., mechanical, such as fungivores, or chemical, like specific growth medium composition [29,43]. Moreover, culture conditions impact the content of metabolites in the exudates [11,34]. For instance, exudates droplets of the *Aspergillus ochraceus* strain accumulated high concentrations of ochratoxin A (OTA) when grown on a wheat-based medium, whereas at malt extract agar OTA was not detected at all [34]. Aliferis and Jabaji studied the overall composition of guttation droplets [17] on *Rhizoctonia solani*, and there are several previous, fragmentary analyses [42,44,45,46]. As a result, the researchers have identified the main components of exudates: SMs, carboxylic acids, carbohydrates, followed by less intensively represented fatty acids and amino acids [17]. Predominantly, chemical characteristics are highly individual, and various conditions induce guttation, which raises questions about the purpose of this phenomenon.

## 3. Ecological Role of Fungal Exudates

The general biological functions of guttation in fungi remain speculative. At times, it is observed that exudate’s droplets are forming in laboratories, conditionally, but not under field conditions, suggesting that, in these cases, they constitute “the image” of secretory activity [15,23,32]. In other cases, especially when fungus has no specific “lifestyle” with the development of specialized ecological relations, we can only suspect some of the general purposes of guttation (e.g., participation in the growth) [14]. However, these general purposes may be equally important; for instance, in young parts of aerial hyphae, threats of desiccation are noticeable, so retaining suitable moisture via exudates should help maintain a constant growth rate, even with unfavorable water potential [14,47]. The same refers to the conception of exudates as a metabolite reservoir, which is supported by the discovery of large amounts of carbohydrates and fatty acids in droplets of some fungi [18,28,34]. Excretion of nutrients collected outside of mycelium, e.g., inositol, mannitol, trehalose, lauric or heptadecanoic acid, is expected to regulate internal physiological mechanisms, and accompany the development of structures, such as sclerotia [42,48]. Therefore, guttation seems to be an important step in the colony maturation process, e.g., linked to cell death [12,13]. Another hypothesis is that fungi remove metabolic byproducts by their secretion, which partially explains a complex composition of exudates, illustrated in biochemical investigations [8,12,18,34].

The idea of exudates as a reservoir of SMs is attracting much more attention due to diverse and specific activities of the metabolites secreted in them [1]. Moreover, identification of SMs and their bioactivities could explain the importance of exudates for the producing strain. Hence, a convincing role of exudates from an entomopathogen, *Metarhizium anisopliae*, was demonstrated by Hutwimmer et al. [15]. The presence of insecticidal destruxins in *Metarhizium* droplets informs researchers about the mechanism of pathogenesis; namely, poisonous fluid is exuded during infection into the host, resulting in its death, which facilitates ingrowth of the fungus [15,49]. The ecological role of exudate droplets, produced by phytopathogenic fungi, such as *Fusarium culmorum* and *Sclerotinia sclerotiorum*, is quite clear. Their exudates, containing lytic enzymes, likely help with invasion and development in the plant [10,12]. The enzymes present in fungal exudates are covered in more detail in Section 5.

Furthermore, virulence and defense reactions are shaped by substances secreted in the exudates. For instance, *Aspergillus nidulans*, after being exposed to prolonged grazing by the fungivore *Collembola*, intensifies guttation and biosynthesis of toxic SMs to discourage further foraging. This likely occurs in response to mechanic hyphae damages or chemical signals [43,50]. Complementing previous suspicions, it is worth noting that, in this case, guttation ran in parallel with the formation of sexual fruiting bodies, called cleistothecia [43]. Likewise, Pandey et al. [37] assumed that SM microfilm, growing around sclerotia of *Sclerotium rolfsii* after drying off the exudates, had a protective function, as it counteracted the degradation of these structures by microorganisms, and supported their survival in soil [37]. Another example of an inducible defensive strategy showed *Xylaria cubensis* FLe9. The FLe9 strain did not exhibit guttation activity in standard culture conditions, whereas after the addition of antifungal amphotericin B to the growth medium, it started to exude mycelial guttates, containing fungistatic metabolites: griseofulvin and dechlorogriseofulvin [29]. Because the microorganisms commonly interact with each other via metabolic exchange and their metabolites act as mediators of many interactions, it is suspected that amphotericin B, being a microbial product present in nature, simulated the presence of a competitor. Such a chemical signal induced a response that, in vivo, helped to outcompete other microorganisms [28,29].

Specific interactions are not always caused by SMs. For example, in *Trichoderma guizhouense* and *Fusarium oxysporum* co-culture, oxidative stress alone might be enough to exert the effect [26]. The growing *Trichoderma* colony extrudes droplets with a large amount of hydrogen peroxide, generated due to the activity of NADPH oxidase and a short-chain dehydrogenase/reductase TgSDR1. This results in poisoning the competitor, which leads to the overgrowth of developmentally arrested *F. oxysporum* [26,51]. Interestingly, the strongest guttation was noticed near to colonies’ contact zone [26]. An analogous situation occurred in the culture of *Pseudoxylaria* sp., paired with other fungal species, where an enhanced exudation rate of the droplets was observed in proximity to the competitor. Moreover, the exudates showed an arsenal of not-yet fully identified SMs [27]. As we can see, more specialized guttation roles often emerge directly from certain SM biological properties (Table 1). In spite of that, some activities that are not connected to the fungus environment, such as antineoplastic, seem to be accidental “side effects”, while these more obvious simply take part in the formation of ecological relationships.

## 4. Secondary Metabolites Present in Fungal Exudates

### 4.1. Antimicrobials

Filamentous fungi tend to be a nearly inexhaustible source of antimicrobial compounds due to their participation in various antagonistic interspecific interactions [1,52]. In this context, the guttation is an attractive “selector” of enormous fungal metabolome, as exudate droplets can serve, in vivo, as a tool in such environmental reactions [19,24,42].

#### 4.1.1. Antibacterial and Antiviral Compounds

There is a strong need for new, biological antimicrobials, because of the increasing resistance to antibiotics and antimicrobial agents among pathogens [53]. In the past few decades, the number of multidrug-resistant bacterial strains noticeably increased, while at the same time, the range of effective antibiotics introduced to the market diminished [53,54,55]. Indeed, prior to the approval of the first siderophore-cephalosporin conjugate in 2020, the latest, previously uncovered class of antibiotics was lipopeptides, represented by daptomycin, discovered in 1986 [53,55]. However, secondary metabolism of environmental microorganisms is a potential source of new drugs, as demonstrated by the discovery of the peptide-like teixobactin [53,56].

**Figure 3:**
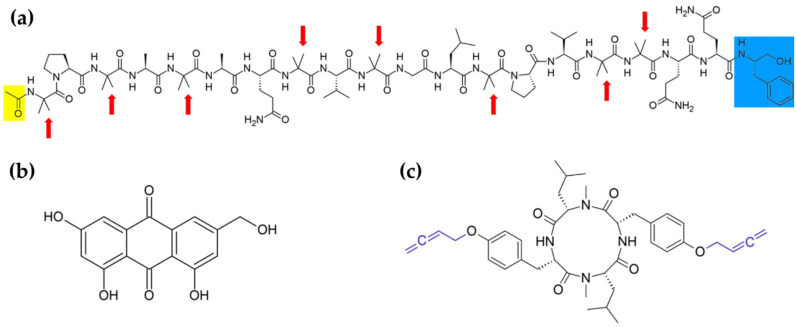
Antimicrobial compounds of the exudates. Molecular structures of: (**a**) peptaibols—on the example of alamethicin F-50 (acetyl group marked in yellow; aminoalcohol marked in blue; red arrows indicate α-aminoisobutyrate residues) [58,60]; (**b**) polyhydroxyanthraquinones—ω-hydroxyemodin [18]; (**c**) pseudoxylallemycins—derivative B (the homoallenyl moieties marked in purple) [27].

One of the most potent groups of antibacterials seems to be the peptaibols family, members of which have been found in fungal exudates [19,57]. The peptaibols are short, linear peptides, containing between 5 and 20 residues, enriched in non-proteinogenic amino acids, such as α-aminoisobutyrate and isovaline [57,58]. The N-terminal group is usually acetylated, and the C-terminus ends with aminoalcohol: mostly phenylalaninol, but seldom valinol, leucinol, isoleucinol, or tryptophanol [58]. A typical molecular structure of these compounds presents alamethicin—the first isolated and most extensively studied peptaibol (Figure 3a) [57]. Generally, peptaibols belong to a widely represented group of antimicrobial peptides (AMPs), biosynthesized by non-ribosomal peptide synthetases [57,59]. The AMPs are host–defense peptides, produced in bacteria, fungi, plants, and animals, to protect them against foreign attacks [59]. Although the scope of peptaibol producers is wide, their most prominent sources are fungi of the *Trichoderma* genus [57,58]. The biological activity of peptaibols is attributed to the formation of ion channels in cell membranes [58,59]. More precisely, they adopt α-helical conformation and possess an amphipathic nature, which enable self-aggregation into oligomeric channel assemblies, spanning across the lipid bilayer [47,50,51]. Apart from antibacterial properties, peptaibols can display antiviral, antifungal, or cytotoxic activity [51,52,53].

An alternative strategy to search for new antibiotics to combat antibiotic-resistant bacteria is to block the expression of their virulence factors. Since the production of these factors is often controlled by quorum sensing, the agents interfering with that signal cascade are appealing tools for antiviral therapy [61,62]. Figueroa et al. [18] isolated a series of known and new polyhydroxyanthraquinones from the exudates of endophytic fungus, *Penicillium restrictum*. The compounds acted as quorum-sensing inhibitors. They were shown to inhibit a functional accessory gene regulator quorum-sensing system in a clinical isolate of methicillin-resistant *Staphylococcus aureus*, leading to the lack of expression of toxins and exoenzymes required to cause an infection [18,63]. As confirmation, the decrease in δ toxin production was noted [18]. Further experiments on the modes of action were conducted on the derivative, ω-hydroxyemodin (Figure 3b), having a relatively strong suppressive effect. They indicated its direct binding to the response regulator AgrA, which subsequently could not associate to *agr* promoters and indirectly upregulate the expression of virulence factors [63].

**Figure 4:**
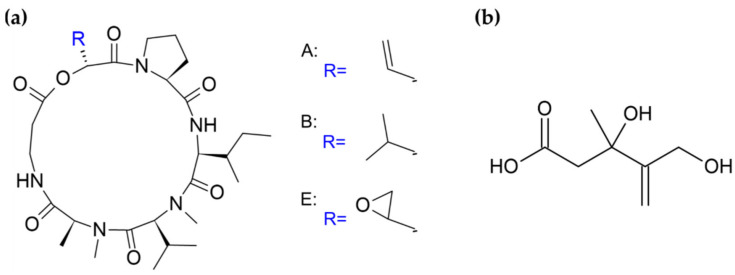
Exuded compounds and agriculture. The structure of: (**a**) destruxins (A, B, E) [70]; (**b**) mevalocidin [71].

Not less interest is connected with search for new antiviral substances. Worth describing here is another AMPs group of compounds found in fungal exudates—destruxins [15]. Similar to peptaibols, the destruxins exhibit a variety of biological activities, some of which will be discussed later. They are cyclic hexadepsipeptides composed of an α-hydroxy acid and five amino acids (Figure 4a) [64]. Individual derivatives differ on the hydroxy acid, N-methylation, and specific R-group patterns of the amino acid residues [64,65]. The main components of *M. anisopliae* exudate droplets are destruxins A, B, and E [15], presented in Figure 4a. In vitro tests implied that some of the destruxins possess anti-hepatitis B activity. Examined derivatives have exerted inhibitory effects on the expression of the hepatitis B surface antigen in human hepatoma cells carrying an integrated viral gene [66,67]. Additionally, destruxins of *Beauveria felina*, along with isaridins and isariins, have been evaluated for anti-Zika virus activities [68]. Some of the isolated compounds revealed inhibitory activity against Zika virus RNA replication and, interestingly, the observed effect in the infected A549 cell line model was stronger than for ivermectin, used due to its high antiviral efficacy as a positive control. Moreover, tested cyclohexadepsipeptides blocked virus entry and downregulated the production of NS5, an essential protein for viral replication [68]. Furthermore, some destruxins are antibacterial agents [69], and possess similar structure pseudoxylallemycins (Figure 3c), which have antibacterial and antineoplastic properties (see Section 4.4) [27].

#### 4.1.2. Antifungal Compounds

Fungitoxic metabolites are recognized as the central points of antagonistic relationships between fungi, e.g., competition or mycoparasitism [4,42,66]. Pandey et al. [37] showed that the exudates collected from *S. rolfsii* had antifungal activity against other plant parasites. The exudates affected spore germination of all 26 tested fungi in vitro and significantly reduced disease incidence in infected plants under field conditions [37]. Chemical analysis of the *S. rolfsii* exudate droplets revealed the presence of phenolic compounds: high concentrations of ferulic acid, as well as tannic, gallic, caffeic, vanillic, chlorogenic, cinnamic, and oxalic acids [37]. Analogically, phenolic compounds turned out to be the major fractions of *R. solani* exudates, with ferulic acid and ethyl ester as the most abundant components [17]. The presence of these compounds contributed to the antifungal effects, since a part of them, e.g., ferulic acid, were reported to be fungitoxic [17,37]. Apart from that, Olsen et al. [38] discovered a new antifungal protein, denoted “*bubble protein*”, in the exudate droplets of *Penicillium brevicompactum* strain (see Section 5). This abundantly exuded constituent of the guttates inhibited the growth of *Saccharomyces cerevisiae* yeasts in a dose-dependent manner [38,39]. Similarly, the *Penicillium chrysogenum* antifungal protein C (PAFC), present in exudates of the Q176 strain, was proven to effectively inhibit *Candida albicans* growth [24].

Experimental reports highlight the importance of the induction of SM biosynthesis gene expression during screening procedures of natural products, particularly in regard to antifungal discovery [2,26,69]. The significance of this was emphasized in the work by Wang et al. [36] on *Penicillium citreonigrum*. The fungus SM profile underwent profound changes in response to chemical epigenetic manipulation [36]. When this Atlantic Forest-soil derived fungus was cultured on an untreated growth medium, it produced colorless exudates containing a simple assemblage of SMs, whereas on a medium with addition of the DNA methyltransferase inhibitor, the exuded droplets became a dark-red color, and were highly enriched in compounds representing distinct biosynthetic families. Six azaphilones and two new meroterpenes were detected. Among them, the sclerotioramine had antifungal properties (it was active against a panel of *Candida* strains) and showed a modest antibacterial effect [36]. Except for one compound, a pencolide, which was detected in both cultures, all others were found exclusively in the exudates of the epigenetically modified fungus [36]. This confirms that epigenetic alterations promote the transcription of silent SM biosynthetic pathways in fungi and impact the metabolite composition of the exudates.

More direct induction was applied in work by Caraballo-Rodríguez et al. [29]. The researchers triggered production and exudation of two antifungal metabolites in *X. cubensis*, by the addition of an antifungal compound to the culture medium [29]. However, both induced metabolites, griseofulvin used as a fungistatic drug, or its derivative dechlorogriseofulvin, are well-known molecules, and were reported earlier in *Xylaria* isolates [72,73]. This includes *X. cubensis* exudates droplets, in which high griseofulvin contents were detected [28]. Sica et al. [28] went a step further in their study, examining *X. cubensis* co-cultured with another fungus instead of introducing a one-compound inductor. The precise influence of the competitor on *X. cubensis* was checked afterwards by Knowles et al. [74]. After growth in co-culture, they established SM profiles of the mycelium surface. As a result, two additional antifungal derivatives of griseofulvin were identified, dechloro-5′-hydroxygriseofulvin and 5′-hydroxygriseofulvin [74]. Importantly, both of these works were aimed at sampling the colony surface, which included exudate chemistry characterization and facilitated dynamic changes monitoring [28,74]. The method, called droplet-based liquid microjunction surface sampling probe, or in short “droplet probe”, was described more broadly in the review by Oberlies et al. [75].

The examples mentioned above do not cover all of the reports. Other works describe antifungal polyene compounds [32,33], unidentified substances that likely grant fungitoxicity to exudates [25,27], or accompanying constituents, such as some fatty acids, that are not discussed here [17].

### 4.2. Bioinsecticides, Bioherbicides

In terms of plant protection, bioinsecticides and bioherbicides are, undoubtedly, significant for agriculture. Efforts made towards their discovery involve the search for new SMs, especially those produced by plant and pest pathogens. This approach is built on the hypothesis that they are the best candidates for biocontrol products [76,77,78]. Some toxins (e.g., mevalocidin) secreted by pathogenic fungi, might present special properties that enable their utilization [71,78]. A few studies on fungal exudates revealed that they accumulated compounds which were implemented successfully. That include both phytotoxins from plant pathogens and toxins of entomopathogenic fungi [15,30].

Secretion of highly insecticidal destruxins by *Metarhizium* or *Beauveria* fungi makes them promising pest biocontrol agents, stimulating their usage [59,79,80,81,82]. The insecticidal activity of destruxins was tested on a variety of pests, proving them to be effective [64]. Insect lethality was a result of the tetanic paralysis and was attributed to opening of ion channels in the plasma membrane. This caused Ca^2+^ influx, which in turn induced muscle depolarization [64,65]. Importantly, both fungi genera are considered safe to human health and environmentally harmless because of neutrality to organisms other than insects [79,80]. These features resulted in extensive exploitation of the strains in agricultural pest control programs, as an alternative to chemical insecticides. *M. anisopliae* or *Beauveria bassiana*-derived formulations had already been registered for commercial use to control tobacco whiteflies, locusts and grasshoppers, spittlebug of sugarcane, red spider mites, thrips, fruit flies, and many others [59,81,82]. Moreover, they may become tools in combating arthropod vectors of human diseases, such as malaria-causing mosquitoes or ticks, a vector of Lyme borreliosis, or tick-borne encephalitis [59,83,84]. The described fungi can be employed in two forms—predominantly, as mycoinsecticide, when their conidia are dispersed, or by application of a mixture of isolated destruxins [81,82].

Mevalocidin is a compound that has a good prospects for its commercial application as a new herbicide. This unique, non-host specific phytotoxin is found in two *Coniolariella* sp. strains and is present in their guttates; it is also actively exuded into the surrounding environment [30,71]. The structure of this toxin (Figure 4b) consists of a pentenoic acid backbone with attached methyl, hydroxy, and hydroxymethyl functional groups. The conformationally-preferred, open chain form exists in equilibrium with the circular, lactone form [30,71]. Mevalocidin has beneficial features. It acts on a wide spectrum of weeds and was observed to cause lethality on all tested grass and broadleaf plant species [71]. It is rapidly absorbed after treatment, achieves a high concentration level, and efficiently translocates through phloem and xylem to other plant parts, including the roots. Moreover, mevalocidin demonstrates broad-spectrum post-emergence activity, stronger than weakly appearing pre-emergence effects, which suggests its rapid degradation in soil. The set of post-emergent symptoms, such as stunting, meristematic inhibition, or anthocyanin accumulation, is unlike those of any commercial herbicide or known phytotoxin, suggesting a novel mode of action [71]. The interference with new targets is an especially valuable feature because weeds become resistant to herbicides, often having the same target sites as older ones [77]. This clearly shows that new biological control agents are needed and that examining fungal guttation may bring real applicative benefits. Additionally, exudates can be potential sources of plant growth and development promoting factors [83,84].

### 4.3. Mycotoxins

Mycotoxins constitute the main health threat among fungal SMs. These compounds, defined as filamentous fungi products, pose health hazards to human and vertebrates by exerting high toxicity on cells [85,86]. Due to the chemical and toxic heterogeneity, mycotoxins are characterized by a wide range of adverse effects, including hepatotoxins, neurotoxins, immunosuppressants, nephrotoxins, hormone analogues (mycohormones), mutagens, carcinogens, or teratogens [86,87].

The pivotal issue connected with mycotoxins is contamination of either farmland or stored crops and food products [86,88]. There are several studies [11,34] on exudates from strains known to biosynthesize OTA, a common toxin of food stock stored in unsuitable conditions [87]. According to Muñoz et al. [34], the *Aspergillus* and *Penicillium* ochratoxigenic strains, cultured on coffee- and wheat-based media, showed different OTA production intensities. Their biomasses contained much more OTA levels in the wheat-based medium than in coffee-based. On wheat-based medium, *Aspergillus* strains formed exudate droplets, which coincided with the high OTA amounts formed by these fungi. The exudate droplets accumulated even higher OTA concentrations than mycelium [34]. Similarly, OTA and ochratoxin B (OTB) level measurements in *Penicillium* strains showed that, in exudates, their content was higher, compared to corresponding mycelia and underlying post-culture medium. The differences reached, respectively, up to 11 and 176 times higher concentrations in exudates than in the mycelium and agar for OTA, and 47.5 and 132 times higher for OTB [11]. The same tendency between the toxicity of exudate droplets and biomass extracts was reported by Salo et al. [20]. Moreover, large amounts of gliotoxin were detected in the exudates of marine-derived *Aspergillus fumigatus* strains [40,41]. Ongoing emissions of this mycotoxin into the seawater can result in its accumulation inside mussels [40]. Infestation of shellfish farming areas by *A. fumigatus* could therefore cause health risks for shellfish consumers [40].

Intake of mycotoxins is also related to their “liberation” into the air. This was studied in *Penicillium expansum*, a strain isolated from indoor building material, by Salo et al. [20]. The strain produced exudate droplets containing chaetoglobosins and communesins. Natural air convection simulating room conditions was induced in the agar Petri dishes, after fungus inoculation, by cooling the dish lids. Both exudate and liquid condensed on the lid were compared, and proven to contain similar patterns of mycotoxins, confirming the transfer of the toxins from exudates into the air. The role of the guttation in a transit of mycotoxins from mold to air cannot be neglected as it is a crucial factor in airborne respiratory toxicity [20,23]. This topic was analyzed deeper on a set of common indoor molds [23]. Metabolites secreted in exudates by isolates of *Aspergillus*, *Chaetomium*, *Penicillium*, *Trichoderma*, *Rhizopus,* and *Stachybotrys* genera were analyzed. Most of the strains, except for *Aspergillus* and *Rhizopus*, emitted mycotoxins in the exudate droplets. Additionally, *T. atroviride*, *Rhizopus,* and *Stachybotrys* sp. released biosurfactants in this way, which may influence the spread of microbial pollutants [23]. Moreover, the high toxicity only referred to the fungal biomass of young, actively growing, and guttating colonies. In comparison, over 6-month-old cultures displayed more than 10 times weaker toxic effects on mammalian cells [23].

There is no clear separation between food and air mycotoxins, rather they are intertwined. One example comes from a macrocyclic trichothecenes producing fungi, such as *Stachybotrys* genus, which can be responsible for serious contamination of crops, food reserves, or buildings [5,89]. These trichothecenes were observed to be secreted into the environment via guttation by some *Stachybotrys chartarum* strains [5]. However, not all of the macrocyclic trichothecenes are harmful to the human organism; they possess additional activities, e.g., antifungal and anticancer [90,91]. On the other hand, mycotoxins can be found among the peptaibols family. A good example involves peptaibols from the exudates of *Trichoderma* strains, isolated from contaminated buildings, where occupants reported indoor air-related disease symptoms [19]. The above examples illustrate that there are SM families with compounds that differ in biological activity, and can exert various effects on humans, from harmful to beneficial, making them interesting objects for further investigations.

### 4.4. Anticancer Substances

It is imperative to establish new, more specific, antineoplastic therapeutics, as cancerous diseases cause critical problems in modern societies. For example, hepatocellular carcinoma (HCC), one of the most malignant and widespread cancers, is the third most frequent cause of cancer-related death worldwide and shows high chemoresistance to many available drugs [92]. Therefore, HCC cells were recently targeted with peptaibols [93,94]. Trichokonin VI was found to suppress growth in the HCC HepG2 line, by the induction of calcium-mediated apoptosis and autophagy [93]. More precisely, it triggered the influx of extracellular calcium, inducing a calpain-dependent, intrinsic mitochondrial pathway [94]. It may be a universal mechanism of peptaibols action, because of their ability to form ion, Ca^2+^-permeable channels in lipid bilayer membranes [94,95].

As the peptaibols family is structurally diverse, trichokonin subclasses have been investigated as well [96]. Anticancer activity against selected cell lines showed also: culicinin D on breast tumor cells [97], alamethicin F50 derivative on a panel of cancer lines [60], emericellipsin on HepG2 [98], and potentially trilongins, as proteasome inhibitors [99]. It is worth noticing that members of different peptaibol subclasses have been found in fungal exudates [19]. Castagnoli et al. [19] identified toxins belonging to several peptaibol groups, such astrichorzianines, trilongins, and trichostrigocin-like peptaibols, in the exudates of various *Trichoderma* strains. In research by Rivera-Chávez et al. [60], aimed at SMs occurring directly on the surface of a fungal culture, new peptaibol derivatives were identified. The research was based on earlier, in situ, searching strategy, enabling surface sampling that included exudates [31,75,100].

The next broad class of anticancer molecules that can be found in exudate droplets are short cyclic peptides. Examples include previously-mentioned pseudoxylallemycins or destruxins [27,70]. Some of the pseudoxylallemycins, tetrapeptides from *Pseudoxylaria* sp. X802 exudates, demonstrated to exhibit antiproliferative activity against human umbilical vein endothelial cells and the K-562 cell line, as well as cytotoxic activity towards HeLa cells [27]. Despite that, the overall molecular mechanism of their anticancer effects remains unknown hitherto. However, their structure characterizations suggested a contribution of rare allenyl modifications in the compound activity [27,101]. Moreover, homoallenyl-tyrosine moieties (Figure 3c) are amenable sites for chemical alterations, enabling easy creation of new derivatives [101].

Interestingly, some compounds among the destruxin mixture exuded by *Metarhizium* fungi have strong antineoplastic properties [70,102,103]. The anticancer action mechanisms of the three most common destruxin derivatives, A, B, and E, have been investigated on colon cancer cell models [70]. All of them were found to cause an imbalance of the cell cycle and cytotoxicity based on intrinsic apoptosis induction, as well as an associated with phosphoinositide-3-kinase/Akt signaling pathway inhibition. Moreover, destruxins inhibit the migration and tube formation of human endothelial cells, which indicates antiangiogenic potential [70]. At the same time, only destruxin E caused intensive disturbance of the intracellular redox balance and showed the strongest antiproliferative activity among them, already at a nanomolar range [70]. In another study, Huynh et al., (2014) confirmed the effectiveness of destruxin B against HCC. In that case, the cell proliferation inhibition was associated mainly with attenuation of the Wnt/β-catenin pathway, fundamental for HCC carcinogenesis and progression [103]. This observation is largely consistent with earlier experiments [102,104] and likely also refers to the E derivative, which exerts anchorage-independent growth inhibition connected with decreased expression of cyclin D1, on the immortalized cell line [102]. Other reports, on different cancer cell lines, highlighted the role of apoptosis induction by destruxin B in investigated cancer cells [105,106,107]. Thus, as the molecular mechanisms behind such compounds vary, both cyclic peptides and linear peptaibols represent promising multifunctional anticancer drug candidates for preclinical development.

## 5. Proteins Excreted by Guttation

The specific properties of fungal exudates could be determined not only by small-molecule SMs—some reports indicate that various proteins are secreted via guttation [10,16]. Jones [108] noticed that *S. sclerotiorum* exudates showed activity of phenol oxidase [108]. Colotelo et al. [42] measured the protein content and presence of selected groups of enzymes in *S. sclerotiorum* exudate. As a result, they additionally confirmed β-glucosidase, catalase, peroxidase, and polyphenoloxidase activities [42]. Furthermore, in exudates of *S. sclerotiorum* and *S. rolfsii*, *P. claviforme* and *F. culmorum* polygalacturonase (pectinase), and cellulolytic activities were detected. Because these fungi are known phytopathogens, observations listed above should be caused by specific enzymes, playing a vital role in pathogenesis by plant tissue lysis [10,42].

Another example of bioactive protein present in fungal exudates constitutes the earlier mentioned *bubble protein* [38,39]. This defensin occurs in high concentrations in the exudates of the *P. brevicompactum* strain. It revealed antifungal effects during inhibition studies [39]. Similar to other members of defensins family, *bubble protein* can be characterized as small-sized protein, enriched in basic amino acids and cysteines. It is stabilized by the framework of disulfide-bridges and mainly consists of beta-sheet structures [39,109]. One general mode of the antimicrobial action of defensins relies on disrupting cell membrane functions by forming channels, resulting in membrane permeabilization, or by modifying membrane transporter activities [109]. High amino acid sequence similarity with the *bubble protein* shares PAFC, one of three PAF proteins that have been isolated from *P. chrysogenum* exudates [24].

A more comprehensive, proteomic study on fungal exudate composition was conducted by Wang et al. [16]. In the exudate of phytopathogenic fungi, *Sclerotinia ginseng*, they found 122 different proteins; 59 of them were identified and classified into six categories: carbohydrate metabolism, oxidation-reduction process, transport and catabolism, amino acid metabolism, proteins performing other functions, and those with unknown roles [16]. Part of the proteins in the carbohydrate metabolism group were associated with the sclerotium development process while the other was involved in virulence. These results are consistent with earlier observations of closely related *S. sclerotiorum* [16,35]. The identified carbohydrate metabolism proteins were, particularly, polysaccharide-degrading enzymes, either acting with fungal specific polymers, such as glucan, or the hydrolyzing plant sugars. The first group is responsible for modifications of a fungal cell wall architecture, whereas the second group catalyzes depolymerization of the plant-host cell wall structural components during invasion [16]. Moreover, in both works, among the detected proteins, those belonging to groups associated with primary metabolism were present, e.g., energy metabolism, but also factors crucial for signal transduction, SM biosynthesis, etc. [16,35].

Recent reports showed even larger numbers of secreted proteins. In exudate droplets of *S. sclerotiorum*, researchers identified a total of 258 proteins [22], in *Cercospora armoraciae*—576 proteins [25], while in exudates of *Ustilaginoidea virens—* 650 various proteins were detected [21]. Proteomic analysis of *S. sclerotiorum* exudates overlapped to some degree with earlier observations—four proteins were recognized as being related to plant cell wall degradation, contributing to host tissue necrosis [22]. Two works by Wang et al. [21,25] reported a number of metabolically relevant protein groups, covering almost the entire process of fungal growth and development, suggesting participation of exudates in the whole strain life cycle. Among the reported proteins, there were cell cycle proteins, ones responsible for signaling, nutrient catabolism, xenobiotics biodegradation, posttranslational modifications, and transport [21,25]. Moreover, many exuded proteins, especially those involved in peroxisome metabolism and biosynthetic pathways, might confer antifungal, antioxidant, and antimicrobial activity upon the exudates [25]. It shows how abundant the fungal exudate proteome is, and how far it impacts the strain features. The exact mechanisms of the protein delivery to the exudates are not known; further research is required. The exudate droplets can either contain proteins characteristic for secretome, whose activity is specifically extracellular, or those connected with intracellular processes [25,35].

## 6. Conclusions

Guttation in fungi occurs at different conditions, and its ecological role is sometimes elusive. At the moment, we can only speculate that guttation is a process occurring from whole mycelium and the droplets are forming on the intersection of environments with different state of matter e.g., solid/liquid and air intersection. On such intersection water evaporates and droplets are formed with higher concentration of metabolites. At the same time in liquid or solid media/environment the exuded substances diffuse or interact with surrounding matter. The wealth of metabolites, possessing specific bioactivities in exudates, is enormous. For example, we can find molecules with antagonistic activities against other organisms, some of which have biotechnological and biomedical potential, i.e., as new antibiotics or antitumor agents. Others display risks, as they can be harmful to humans or live-stock animals. The multitudes of interactions and activities of the exuded compounds are reflected in their structural and chemical diversity. The research discussed here sheds new light on the significance and real nature of the guttation phenomenon. Nevertheless, many aspects of the phenomenon still require research. It would be interesting to see how the composition of the exudates change with culture conditions. Moreover, elucidation of delivery mechanisms, of metabolites and proteins to the exudates, requires more research. It would be of interest to investigate whether the exudate droplets contain extracellular vesicles, as these could be carriers of large varieties of molecules [110]. In our work, we outlined fungal guttation, an interesting (yet, still largely unknown) research subject. We also highlighted its essential importance to the natural product research community and other branches of science.

## Figures and Tables

**Table 1:** Reports on bioactive compounds found in fungal exudates and culture conditions enabling guttation.

Fungus Name	Growth Medium	Incubation T	Identified Compounds	Bioactivity	Reference
*Sclerotinia sclerotiorum*	PDA	25 °C	proteins (amylases, cellulases, hemicellulases, polygalacturonases)	plant cell wall degrading (enzymatic)	[22]
*Ustilaginoidea virens*	PSA	25 ± 1 °C	proteins (acetylxylan esterase, cellobiose dehydrogenase, endo-1,3(4)-beta-glucanase; superoxide dismutase)	plant cell wall degrading (enzymatic);oxidative stress generation	[21]
*Chaetomium globosum*, *Penicillium expansum*, *Stachybotrys* sp., *Trichoderma atroviride*, *Trichoderma trixiae*	MEA	23 ± 1 °C	–	cytotoxic (mycotoxins)	[23]
*Penicillium chrysogenum*	specific	25 °C	proteins (i.e., PAFC)	antifungal	[24]
*Cercospora armoraciae*	PDA	25 °C ± 1 °C	proteins (putative indirect role—SMs biosynthesis enzymes)	antimicrobial, phytotoxic	[25]
*Penicillium expansum*	MEA	23 ± 1 °C	chaetoglobosins, communesins	cytotoxic (mycotoxins)	[20]
*Trichoderma* *guizhouense*	specific (synthetic), coculture	25 °C	hydrogen peroxide	oxidative stress generation (in another fungus)	[26]
*Trichoderma atroviride*, *Trichoderma citrinoviride*, *Trichoderma paraviridescens*, *Trichoderma trixiae*	MEA	22 °C	peptaibols (trichorzianines, trilongins, trichostrigocins, trichostrigocin-like peptaibols)	cytotoxic (mycotoxins)	[19]
*Sclerotinia ginseng*	PDA	20 ± 1 °C	proteins (cellulases, pectinases)	plant cell wall degrading (enzymatic)	[16]
*Pseudoxylaria* sp.	PDA, coculture	RT	pseudoxylallemycins (*n* = 4)	antibacterial, anticancer	[27]
*Xylariacubensis*	MEA; ISP-2 agar + amphotericin B	–; 30 °C	griseofulvin; griseofulvin, dechlorogriseofulvin	antifungal	[28,29]
*Coniolariella* sp.	agar	–	mevalocidin	phytotoxic (bioherbicide)	[30]
*Clohesyomyces* *aquaticus*	–	–	phomopsinone A	antifungal	[31]
*Penicillium restrictum*	PDA	25 °C	polyhydroxyanthraquinones (*n* = 9)	antibacterial (QS inhibitor)	[18]
*Stachybotryschartarum*	MEA	25 °C	macrocyclic trichothecenes (*n* = 6)	cytotoxic (mycotoxins)	[5]
*Epicoccum nigrum*	CYA	25 °C	polyene compounds (epipyrones)	antifungal	[32,33]
*Aspergillus niger*, *Aspergillus ochraceus*	specific (coffee-, wheat-based)	25 °C	ochratoxin A	cytotoxic (mycotoxin)	[34]
*Rhizoctonia solani*	PDA	24 ± 1 °C	phenolic compounds	antifungal, phytotoxic	[17]
*Sclerotinia sclerotiorum*	PDA	22 ± 1 °C	proteins (arabinofuranosidases; cerato-platanin, necrosis inducing protein)	plant cell wall degrading (enzymatic); phytotoxic	[35]
*Metarhiziumanisopliae*	specific (3 synthetic media)	25 °C	destruxins (A, B, E)	insecticidal, phytotoxic, anticancer, etc.	[15]
*Penicillium* *citreonigrum*	specific + 5-azacytidine	25 °C	sclerotioramine; sclerotiorin	antibacterial, antifungal; antibacterial	[36]
*Penicillium nordicum*, *Penicillium verrucosum*	CYA, MEA	25 °C	ochratoxins (A, B)	cytotoxic (mycotoxins)	[11]
*Sclerotium rolfsii*	PDA	25 ± 2 °C	phenolic compounds	antifungal	[37]
*Penicillium brevicompactum*	specific	20 °C	bubble protein (defensin);mycophenolic acid	antifungal; antimicrobial	[38,39]
*Aspergillus fumigatus*	SDA + seawater;DCA + seawater	27 °C	gliotoxin	cytotoxic (mycotoxin)	[40,41]
*Fusarium culmorum*, *Penicillium claviforme*, *Sclerotinia sclerotiorum*, *Sclerotium rolfsii*	PDA; specific (carrot-based, synthetic)	20, 25 °C	proteins (cellulase, polygalacturonase)	plant cell wall degrading (enzymatic)	[10,42]

*n*—number of compounds, QS—quorum sensing, RT—room temperature, “-”—no data; CYA—Czapek yeast extract agar, DCA—dextrose casein agar, ISP-2—International *Streptomyces* Project-2 medium, MEA—malt extract agar, PDA—potato dextrose agar, PSA—potato sucrose agar, SDA—Sabouraud dextrose agar.

## Data Availability

All materials and data connected with this study are available in the paper.

## References

[1] Bills G.F., Gloer J.B. (2016). Biologically active secondary metabolites from the fungi. Microbiol. Spectr..

[2] Keller N.P. (2019). Fungal secondary metabolism: Regulation, function and drug discovery. Nat. Rev. Microbiol..

[3] Williams D.H., Stone M.J., Hauck P.R., Rahman S.K. (1989). Why are secondary metabolites (natural products) biosynthesized?. J. Nat. Prod..

[4] Macheleidt J., Mattern D.J., Fischer J., Netzker T., Weber J., Schroeckh V., Valiante V., Brakhage A.A. (2016). Regulation and role of fungal secondary metabolites. Annu. Rev. Genet..

[5] Gareis M., Gottschalk C. (2014). *Stachybotrys* spp. and the guttation phenomenon. Mycotoxin Res..

[6] Bayram Ö. (2017). Fungal Jewels: Secondary Metabolites.

[7] Ivanoff S.S. (1963). Guttation injuries of plants. Bot. Rev..

[8] Sun Y.-P., Unestam T., Lucas S.D., Johanson K.J., Kenne L., Finlay R. (1999). Exudation-reabsorption in a mycorrhizal fungus, the dynamic interface for interaction with soil and soil microorganisms. Mycorrhiza.

[9] Thom C. (1930). The Penicillia.

[10] Colotelo N. (1978). Fungal exudates. Can. J. Microbiol..

[11] Gareis M., Gareis E.-M. (2007). Guttation droplets of *Penicillium nordicum* and *Penicillium verrucosum* contain high concentrations of the mycotoxins ochratoxin A and B. Mycopathologia.

[12] McPhee W., Colotelo N. (1977). Fungal exudates. I. Characteristics of hyphal exudates in *Fusarium culmorum*. Can. J. Bot..

[13] Georgiou C.D., Patsoukis N., Papapostolou I., Zervoudakis G. (2006). Sclerotial metamorphosis in filamentous fungi is induced by oxidative stress. Integr. Comp. Biol..

[14] Jennings D.H. (1991). The role of droplets in helping to maintain a constant growth rate of aerial hyphae. Mycol. Res..

[15] Hutwimmer S., Wang H., Strasser H., Burgstaller W. (2010). Formation of exudate droplets by *Metarhizium anisopliae* and the presence of destruxins. Mycologia.

[16] Wang D., Fu J.F., Zhou R.J., Li Z.B., Xie Y.J. (2017). Proteomics research and related functional classification of liquid sclerotial exudates of *Sclerotinia ginseng*. PeerJ.

[17] Aliferis K.A., Jabaji S. (2010). Metabolite composition and bioactivity of *Rhizoctonia solani* sclerotial exudates. J. Agric. Food Chem..

[18] Figueroa M., Jarmusch A.K., Raja H.A., El-Elimat T., Kavanaugh J.S., Horswill A.R., Cooks R.G., Cech N.B., Oberlies N.H. (2014). Polyhydroxyanthraquinones as quorum sensing inhibitors from the guttates of *Penicillium restrictum* and their analysis by desorption electrospray ionization mass spectrometry. J. Nat. Prod..

[19] Castagnoli E., Marik T., Mikkola R., Kredics L., Andersson M.A., Salonen H., Kurnitski J. (2018). Indoor *Trichoderma* strains emitting peptaibols in guttation droplets. J. Appl. Microbiol..

[20] Salo M.J., Marik T., Mikkola R., Andersson M.A., Kredics L., Salonen H., Kurnitski J. (2019). *Penicillium expansum* strain isolated from indoor building material was able to grow on gypsum board and emitted guttation droplets containing chaetoglobosins and communesins A, B and D. J. Appl. Microbiol..

[21] Wang H., Yang X., Wei S., Wang Y. (2021). Proteomic analysis of mycelial exudates of *Ustilaginoidea virens*. Pathogens.

[22] Tian J., Chen C., Sun H., Wang Z., Steinkellner S., Feng J., Liang Y. (2021). Proteomic analysis reveals the importance of exudates on sclerotial development in *Sclerotinia sclerotiorum*. J. Agric. Food Chem..

[23] Andersson M.A., Salo J., Kedves O., Kredics L., Druzhinina I., Kurnitski J., Salonen H. (2020). Bioreactivity, guttation and agents influencing surface tension of water emitted by actively growing indoor mould isolates. Microorganisms.

[24] Holzknecht J., Kühbacher A., Papp C., Farkas A., Váradi G., Marcos J.F., Manzanares P., Tóth G.K., Galgóczy L., Marx F. (2020). The *Penicillium chrysogenum* Q176 antimicrobial protein PAFC effectively inhibits the growth of the opportunistic human pathogen *Candida albicans*. J. Fungi.

[25] Wang H., Wei S., Yang X., Liu W., Zhu L. (2020). Proteomic analysis of exudate of *Cercospora armoraciae* from *Armoracia rusticana*. PeerJ.

[26] Zhang J., Miao Y., Rahimi M.J., Zhu H., Steindorff A., Schiessler S., Cai F., Pang G., Chenthamara K., Xu Y. (2019). Guttation capsules containing hydrogen peroxide: An evolutionarily conserved NADPH oxidase gains a role in wars between related fungi. Environ. Microbiol..

[27] Guo H., Kreuzenbeck N.B., Otani S., Garcia-Altares M., Dahse H.-M., Weigel C., Aanen D.K., Hertweck C., Poulsen M., Beemelmanns C. (2016). Pseudoxylallemycins A-F, cyclic tetrapeptides with rare allenyl modifications isolated from *Pseudoxylaria* sp. X802: A competitor of fungus-growing termite cultivars. Org. Lett..

[28] Sica V.P., Rees E.R., Tchegnon E., Bardsley R.H., Raja H.A., Oberlies N.H. (2016). Spatial and temporal profiling of griseofulvin production in *Xylaria cubensis* using mass spectrometry mapping. Front. Microbiol..

[29] Caraballo-Rodríguez A.M., Mayor C.A., Chagas F.O., Pupo M.T. (2017). Amphotericin B as an inducer of griseofulvin-containing guttate in the endophytic fungus *Xylaria cubensis* FLe9. Chemoecology.

[30] Sica V.P., Figueroa M., Raja H.A., El-Elimat T., Darveaux B.A., Pearce C.J., Oberlies N.H. (2016). Optimizing production and evaluating biosynthesis *in situ* of a herbicidal compound, mevalocidin, from *Coniolariella* sp.. J. Ind. Microbiol. Biotechnol..

[31] Sica V.P., Raja H.A., El-Elimat T., Kertesz V., Van Berkel G.J., Pearce C.J., Oberlies N.H. (2015). Dereplicating and spatial mapping of secondary metabolites from fungal cultures in situ. J. Nat. Prod..

[32] Calder C., Ford S., Selwood A.I., Ginkel R.V., Wilkins A.L. (2012). Anti-Microbial Compositions. WO Patent.

[33] Lee A.J., Cadelis M.M., Kim S.H., Swift S., Copp B.R., Villas-Boas S.G. (2020). Epipyrone A, a broad-spectrum antifungal compound produced by *Epicoccum nigrum* ICMP 19927. Molecules.

[34] Muñoz K., Vega M., Rios G., Geisen R., Degen G.H. (2011). Mycotoxin production by different ochratoxigenic *Aspergillus* and *Penicillium* species on coffee- and wheat-based media. Mycotoxin Res..

[35] Liang Y., Strelkov S.E., Kav N.N.V. (2010). The proteome of liquid sclerotial exudates from *Sclerotinia sclerotiorum*. J. Proteome Res..

[36] Wang X., Sena Filho J.G., Hoover A.R., King J.B., Ellis T.K., Powell D.R., Cichewicz R.H. (2010). Chemical epigenetics alters the secondary metabolite composition of guttate excreted by an atlantic-forest-soil-derived *Penicillium citreonigrum*. J. Nat. Prod..

[37] Pandey M.K., Sarma B.K., Singh D.P., Singh U.P. (2007). Biochemical investigations of sclerotial exudates of *Sclerotium rolfsii* and their antifungal activity. J. Phytopathol..

[38] Olsen J.G., Flensburg C., Olsen O., Bricogne G., Henriksen A. (2004). Solving the structure of the bubble protein using the anomalous sulfur signal from single-crystal in-house Cu *K*α diffraction data only. Acta Crystallogr. D Biol. Crystallogr..

[39] Seibold M., Wolschann P., Bodevin S., Olsen O. (2011). Properties of the bubble protein, a defensin and an abundant component of a fungal exudate. Peptides.

[40] Grovel O., Pouchus Y.F., Verbist J.-F. (2003). Accumulation of gliotoxin, a cytotoxic mycotoxin from *Aspergillus fumigatus*, in blue mussel (*Mytilus edulis*). Toxicon.

[41] Kerzaon I., Grovel O., Robiou Du Pont T., Le Pape P., Pouchus Y.-F. (2008). Effects of seawater on growth and gliotoxin excretion of marine strains of *Aspergillus fumigatus* Fres. Toxicon Off. J. Int. Soc. Toxinol..

[42] Colotelo N., Sumner J.L., Voegelin W.S. (1971). Chemical studies on the exudate and developing sclerotia of *Sclerotinia sclerotiorum* (Lib.) DeBary. Can. J. Microbiol..

[43] Döll K., Chatterjee S., Scheu S., Karlovsky P., Rohlfs M. (2013). Fungal metabolic plasticity and sexual development mediate induced resistance to arthropod fungivory. Proc. Biol. Sci..

[44] Colotelo N. (1973). Physiological and biochemical properties of the exudate associated with developing sclerotia of *Sclerotinia sclerotiorum* (Lib.) DeBary. Can. J. Microbiol..

[45] Reddy M.N., Anandaraj G.V.D., Devi P.S. (1989). Chemical composition of sclerotial exudates of *Macrophomina phaseolina* pathogenic to groundnut. Soil Biol. Biochem..

[46] Sarma B.K., Singh U.P., Singh K.P. (2002). Variability in Indian isolates of *Sclerotium rolfsii*. Mycologia.

[47] Coggins C.R., Jennings D.H., Clarke R.W. (1980). Tear or drop formation by mycelium of *Serpula lacrimans*. Trans. Br. Mycol. Soc..

[48] Cooke R.C. (1969). Changes in soluble carbohydrates during sclerotium formation by *Sclerotinia sclerotiorum* and *S. trifoliorum*. Trans. Br. Mycol. Soc..

[49] Ríos-Moreno A., Garrido-Jurado I., Raya-Ortega M.C., Quesada-Moraga E. (2017). Quantification of fungal growth and destruxin A during infection of *Galleria mellonella* larvae by *Metarhizium brunneum*. J. Invertebr. Pathol..

[50] Rohlfs M. (2014). Fungal secondary metabolite dynamics in fungus-grazer interactions: Novel insights and unanswered questions. Front. Microbiol..

[51] Zhu H., Zhang J., Gao Q., Pang G., Sun T., Li R., Yu Z., Shen Q. (2021). A new atypical short-chain dehydrogenase is required for interfungal combat and conidiation in *Trichoderma guizhouense*. Environ. Microbiol..

[52] Karwehl S., Stadler M. (2016). Exploitation of fungal biodiversity for discovery of novel antibiotics. Curr. Top. Microbiol. Immunol..

[53] Durand G.A., Raoult D., Dubourg G. (2019). Antibiotic discovery: History, methods and perspectives. Int. J. Antimicrob. Agents.

[54] Van Duin D., Paterson D.L. (2020). Multidrug-resistant bacteria in the community: An update. Infect. Dis. Clin. N. Am..

[55] Terreni M., Taccani M., Pregnolato M. (2021). New antibiotics for multidrug-resistant bacterial strains: Latest research developments and future perspectives. Molecules.

[56] Ling L.L., Schneider T., Peoples A.J., Spoering A.L., Engels I., Conlon B.P., Mueller A., Schäberle T.F., Hughes D.E., Epstein S. (2015). A new antibiotic kills pathogens without detectable resistance. Nature.

[57] Duclohier H. (2010). Antimicrobial peptides and peptaibols, substitutes for conventional antibiotics. Curr. Pharm. Des..

[58] Daniel J.F.S., Filho E.R. (2007). Peptaibols of *Trichoderma*. Nat. Prod. Rep..

[59] Lei J., Sun L., Huang S., Zhu C., Li P., He J., Mackey V., Coy D.H., He Q. (2019). The antimicrobial peptides and their potential clinical applications. Am. J. Transl. Res..

[60] Rivera-Chávez J., Raja H.A., Graf T.N., Gallagher J.M., Metri P., Xue D., Pearce C.J., Oberlies N.H. (2017). Prealamethicin F50 and related peptaibols from *Trichoderma arundinaceum*: Validation of their authenticity via in situ chemical analysis. RSC Adv..

[61] Dickey S.W., Cheung G.Y.C., Otto M. (2017). Different drugs for bad bugs: Antivirulence strategies in the age of antibiotic resistance. Nat. Rev. Drug Discov..

[62] Defoirdt T. (2018). Quorum-sensing systems as targets for antivirulence therapy. Trends Microbiol..

[63] Daly S.M., Elmore B.O., Kavanaugh J.S., Triplett K.D., Figueroa M., Raja H.A., El-Elimat T., Crosby H.A., Femling J.K., Cech N.B. (2015). ω-Hydroxyemodin limits *Staphylococcus aureus* quorum sensing-mediated pathogenesis and inflammation. Antimicrob. Agents Chemother..

[64] Pedras M.S.C., Irina Zaharia L., Ward D.E. (2002). The destruxins: Synthesis, biosynthesis, biotransformation, and biological activity. Phytochemistry.

[65] Liu B.-L., Tzeng Y.-M. (2012). Development and applications of destruxins: A review. Biotechnol. Adv..

[66] Yeh S.F., Pan W., Ong G.T., Chiou A.J., Chuang C.C., Chiou S.H., Wu S.H. (1996). Study of structure-activity correlation in destruxins, a class of cyclodepsipeptides possessing suppressive effect on the generation of hepatitis B virus surface antigen in human hepatoma cells. Biochem. Biophys. Res. Commun..

[67] Chen H.C., Chou C.K., Sun C.M., Yeh S.F. (1997). Suppressive effects of destruxin B on hepatitis B virus surface antigen gene expression in human hepatoma cells. Antivir. Res..

[68] Yuan B., Wu Z., Ji W., Liu D., Guo X., Yang D., Fan A., Jia H., Ma M., Lin W. (2021). Discovery of cyclohexadepsipeptides with anti-Zika virus activities and biosynthesis of the nonproteinogenic building block (3S)-methyl-l-proline. J. Biol. Chem..

[69] Che Y., Swenson D.C., Gloer J.B., Koster B., Malloch D. (2001). Pseudodestruxins A and B: New cyclic depsipeptides from the coprophilous fungus *Nigrosabulum globosum*. J. Nat. Prod..

[70] Dornetshuber-Fleiss R., Heffeter P., Mohr T., Hazemi P., Kryeziu K., Seger C., Berger W., Lemmens-Gruber R. (2013). Destruxins: Fungal-derived cyclohexadepsipeptides with multifaceted anticancer and antiangiogenic activities. Biochem. Pharmacol..

[71] Gerwick B.C., Brewster W.K., Deboer G.J., Fields S.C., Graupner P.R., Hahn D.R., Pearce C.J., Schmitzer P.R., Webster J.D. (2013). Mevalocidin: A novel, phloem mobile phytotoxin from *Fusarium* DA056446 and *Rosellinia* DA092917. J. Chem. Ecol..

[72] Park J.H., Choi G.J., Lee H.B., Kim K.M., Jung H.S., Lee S.W., Jang K.S., Cho K.Y., Kim J.C. (2005). Griseofulvin from *Xylaria* sp. strain F0010, an endophytic fungus of *Abies holophylla* and its antifungal activity against plant pathogenic fungi. J. Microbiol. Biotechnol..

[73] Brooks W.C., Paguigan N.D., Raja H.A., Moy F.J., Cech N.B., Pearce C.J., Oberlies N.H. (2017). qNMR for profiling the production of fungal secondary metabolites. Magn. Reson. Chem. MRC.

[74] Knowles S.L., Raja H.A., Wright A.J., Lee A.M.L., Caesar L.K., Cech N.B., Mead M.E., Steenwyk J.L., Ries L.N.A., Goldman G.H. (2019). Mapping the fungal battlefield: Using *in situ* chemistry and deletion mutants to monitor interspecific chemical interactions between fungi. Front. Microbiol..

[75] Oberlies N.H., Knowles S.L., Amrine C.S.M., Kao D., Kertesz V., Raja H.A. (2019). Droplet probe: Coupling chromatography to the in situ evaluation of the chemistry of nature. Nat. Prod. Rep..

[76] Sudakin D.L. (2003). Biopesticides. Toxicol. Rev..

[77] Dayan F.E., Owens D.K., Duke S.O. (2012). Rationale for a natural products approach to herbicide discovery. Pest Manag. Sci..

[78] Lacey L.A., Grzywacz D., Shapiro-Ilan D.I., Frutos R., Brownbridge M., Goettel M.S. (2015). Insect pathogens as biological control agents: Back to the future. J. Invertebr. Pathol..

[79] Mascarin G.M., Jaronski S.T. (2016). The production and uses of *Beauveria bassiana* as a microbial insecticide. World J. Microbiol. Biotechnol..

[80] Stone L.B.L., Bidochka M.J. (2020). The multifunctional lifestyles of *Metarhizium*: Evolution and applications. Appl. Microbiol. Biotechnol..

[81] Lozano-Tovar M.D., Garrido-Jurado I., Lafont F., Quesada-Moraga E. (2015). Insecticidal activity of a destruxin-containing extract of *Metarhizium brunneum* against *Ceratitis capitata* (Diptera: Tephritidae). J. Econ. Entomol..

[82] Oliveira D.G.P., Pauli G., Mascarin G.M., Delalibera I. (2015). A protocol for determination of conidial viability of the fungal entomopathogens *Beauveria bassiana* and *Metarhizium anisopliae* from commercial products. J. Microbiol. Methods.

[83] Calcagno C., Novero M., Genre A., Bonfante P., Lanfranco L. (2012). The exudate from an arbuscular mycorrhizal fungus induces nitric oxide accumulation in *Medicago truncatula* roots. Mycorrhiza.

[84] Vujanovic V., Germida J.J. (2019). Endophytic Microbial Symbionts in Plant Prenatal Care 2019. U.S. Patent.

[85] Taevernier L., Wynendaele E., De Vreese L., Burvenich C., De Spiegeleer B. (2016). The mycotoxin definition reconsidered towards fungal cyclic depsipeptides. J. Environ. Sci. Health Part C Environ. Carcinog. Ecotoxicol. Rev..

[86] Pleadin J., Frece J., Markov K., Toldrá F. (2019). Mycotoxins in food and feed. Advances in Food and Nutrition Research.

[87] Richard J.L. (2007). Some major mycotoxins and their mycotoxicoses–An overview. Int. J. Food Microbiol..

[88] Foroud N.A., Eudes F. (2009). Trichothecenes in cereal grains. Int. J. Mol. Sci..

[89] McCormick S.P., Stanley A.M., Stover N.A., Alexander N.J. (2011). Trichothecenes: From simple to complex mycotoxins. Toxins.

[90] De Carvalho M.P., Weich H., Abraham W.-R. (2016). Macrocyclic trichothecenes as antifungal and anticancer compounds. Curr. Med. Chem..

[91] Zhu M., Cen Y., Ye W., Li S., Zhang W. (2020). Recent advances on macrocyclic trichothecenes, their bioactivities and biosynthetic pathway. Toxins.

[92] Forner A., Llovet J.M., Bruix J. (2012). Hepatocellular carcinoma. Lancet Lond. Engl..

[93] Shi M., Wang H.-N., Xie S.-T., Luo Y., Sun C.-Y., Chen X.-L., Zhang Y.-Z. (2010). Antimicrobial peptaibols, novel suppressors of tumor cells, targeted calcium-mediated apoptosis and autophagy in human hepatocellular carcinoma cells. Mol. Cancer.

[94] Shi M., Zhang T., Sun L., Luo Y., Liu D.-H., Xie S.-T., Song X.-Y., Wang G.-F., Chen X.-L., Zhou B.-C. (2013). Calpain, Atg5 and Bak play important roles in the crosstalk between apoptosis and autophagy induced by influx of extracellular calcium. Apoptosis Int. J. Program. Cell Death.

[95] Chugh J.K., Wallace B.A. (2001). Peptaibols: Models for ion channels. Biochem. Soc. Trans..

[96] Marik T., Tyagi C., Balázs D., Urbán P., Szepesi Á., Bakacsy L., Endre G., Rakk D., Szekeres A., Andersson M.A. (2019). Structural diversity and bioactivities of peptaibol compounds from the Longibrachiatum Clade of the filamentous fungal genus *Trichoderma*. Front. Microbiol..

[97] He H., Janso J.E., Yang H.Y., Bernan V.S., Lin S.L., Yu K. (2006). Culicinin D, an antitumor peptaibol produced by the fungus *Culicinomyces clavisporus*, strain LL-12I252. J. Nat. Prod..

[98] Rogozhin E.A., Sadykova V.S., Baranova A.A., Vasilchenko A.S., Lushpa V.A., Mineev K.S., Georgieva M.L., Kul’ko A.B., Krasheninnikov M.E., Lyundup A.V. (2018). A novel lipopeptaibol emericellipsin A with antimicrobial and antitumor activity produced by the extremophilic fungus *Emericellopsis alkalina*. Molecules.

[99] Grigoletto D.F., Trivella D.B.B., Tempone A.G., Rodrigues A., Correia A.M.L., Lira S.P. (2020). Antifungal compounds with anticancer potential from *Trichoderma* sp. P8BDA1F1, an endophytic fungus from *Begonia venosa*. Braz. J. Microbiol. Publ. Braz. Soc. Microbiol..

[100] Sica V.P., Rees E.R., Raja H.A., Rivera-Chávez J., Burdette J.E., Pearce C.J., Oberlies N.H. (2017). *In situ* mass spectrometry monitoring of fungal cultures led to the identification of four peptaibols with a rare threonine residue. Phytochemistry.

[101] Guo H., Schmidt A., Stephan P., Raguž L., Braga D., Kaiser M., Dahse H.-M., Weigel C., Lackner G., Beemelmanns C. (2018). Precursor-directed diversification of cyclic tetrapeptidic pseudoxylallemycins. Chembiochem. Eur. J. Chem. Biol..

[102] Kobayashi T., Ikeno S., Hosokawa N., Uehara Y., Hori M., Tsuchiya K. (2004). Destruxin E, a cyclodepsipeptide antibiotic, reduces cyclin D1 levels and inhibits anchorage-independent growth of v-Ki-ras-expressed pMAM-ras-REF cells. Biol. Pharm. Bull..

[103] Huynh T.-T., Rao Y.K., Lee W.-H., Chen H.-A., Le T.D.-Q., Tzeng D.T.W., Wang L.-S., Wu A.T.H., Lin Y.-F., Tzeng Y.-M. (2014). Destruxin B inhibits hepatocellular carcinoma cell growth through modulation of the Wnt/β-catenin signaling pathway and epithelial-mesenchymal transition. Toxicol. In Vitro Int. J. Publ. Assoc. BIBRA.

[104] Yeh C.-T., Rao Y.K., Ye M., Wu W.-S., Chang T.-C., Wang L.-S., Wu C.-H., Wu A.T.H., Tzeng Y.-M. (2012). Preclinical evaluation of destruxin B as a novel Wnt signaling target suppressing proliferation and metastasis of colorectal cancer using non-invasive bioluminescence imaging. Toxicol. Appl. Pharmacol..

[105] Lee Y.-P., Wang C.-W., Liao W.-C., Yang C.-R., Yeh C.-T., Tsai C.-H., Yang C.-C., Tzeng Y.-M. (2012). *In vitro* and *in vivo* anticancer effects of destruxin B on human colorectal cancer. Anticancer Res..

[106] Wu C.-C., Chen T.-H., Liu B.-L., Wu L.-C., Chen Y.-C., Tzeng Y.-M., Hsu S.-L. (2013). Destruxin B isolated from entomopathogenic fungus *Metarhizium anisopliae* induces apoptosis via a Bcl-2 family-dependent mitochondrial pathway in human nonsmall cell lung cancer cells. Evid. Based Complement. Altern. Med. ECAM.

[107] Huang Liu R., Chen S.-P., Lu T.-M., Tsai W.-Y., Tsai C.-H., Yang C.-C., Tzeng Y.-M. (2014). Selective apoptotic cell death effects of oral cancer cells treated with destruxin B. BMC Complement. Altern. Med..

[108] Jones D. (1970). Ultrastructure and composition of the cell walls of *Sclerotinia sclerotiorum*. Trans. Br. Mycol. Soc..

[109] Wong J.H., Xia L., Ng T.B. (2007). A review of defensins of diverse origins. Curr. Protein Pept. Sci..

[110] Wolf J.M., Espadas J., Luque-Garcia J., Reynolds T., Casadevall A. (2015). Lipid biosynthetic genes affect *Candida albicans* extracellular vesicle morphology, cargo, and immunostimulatory properties. Eukaryot. Cell.

